# Supplementary material for: Extinct type of human parvovirus B19 persists in tonsillar B cells
Source: Nat Commun. 2017 Apr 4;8:14930. doi: 10.1038/ncomms14930 (PMC5382274; doi:10.1038/ncomms14930)
Supplement: Supplementary Information — Supplementary Figures and Supplementary Tables [file ncomms14930-s1.pdf]

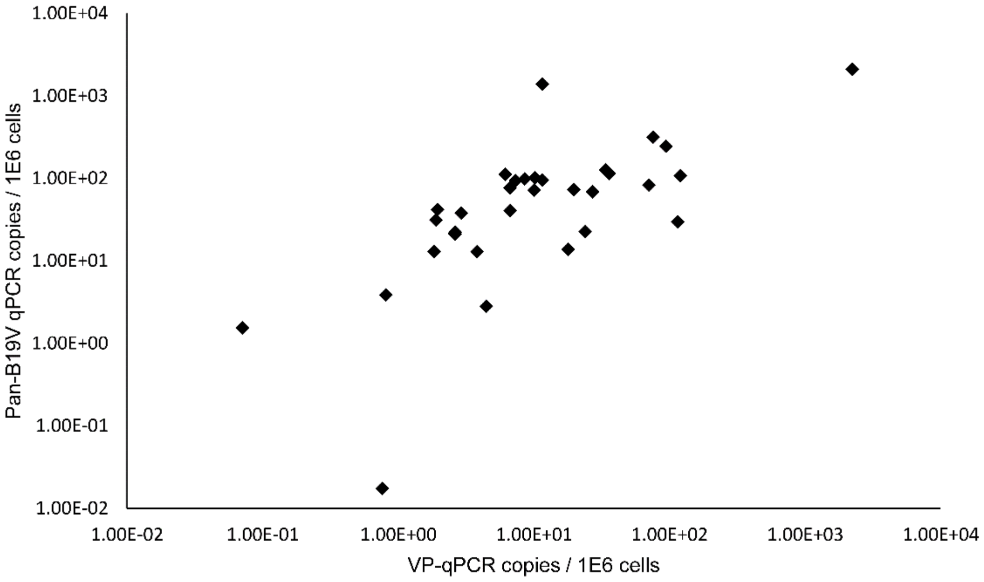

Supplementary Figure 1. B19V loads determined by Pan-B19V-qPCR and VP-qPCR. The B19V-DNA copy numbers in B cells of collagenase treated tonsils (n=33) were determined with two qPCRs targeting distinct genomic regions (NS1 and VP1) and normalized to cell numbers with the human single copy gene *RNase P*.

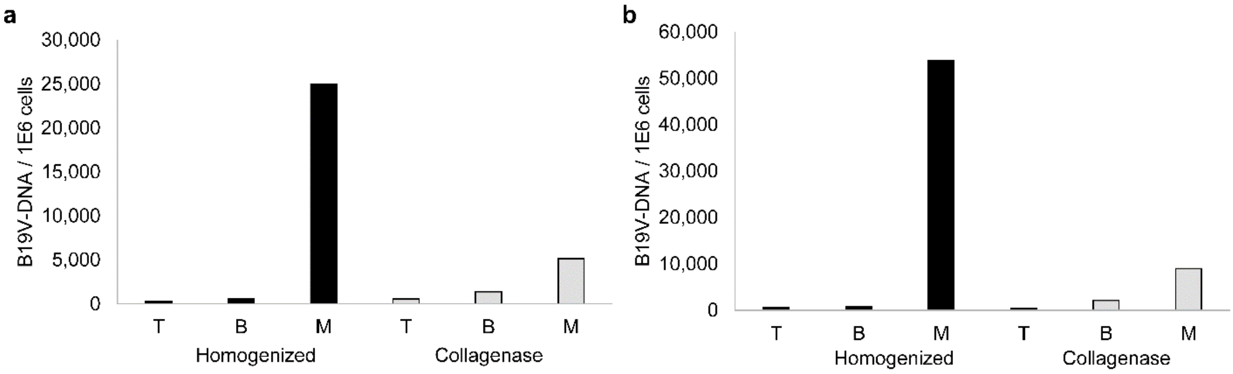

Supplementary Figure 2. B19V-DNA quantities in lymphoid fractions from two children.

B19V-DNA copies in T, B and monocytic cells following homogenization (black) or collagenase digestion (light grey) in a 6-year old **(a)** and an 8-year old patient **(b)** with the highest copy number in the cohort. Represented are B19V-DNA copies normalized to cell numbers with the human single-copy gene *RNase P*.

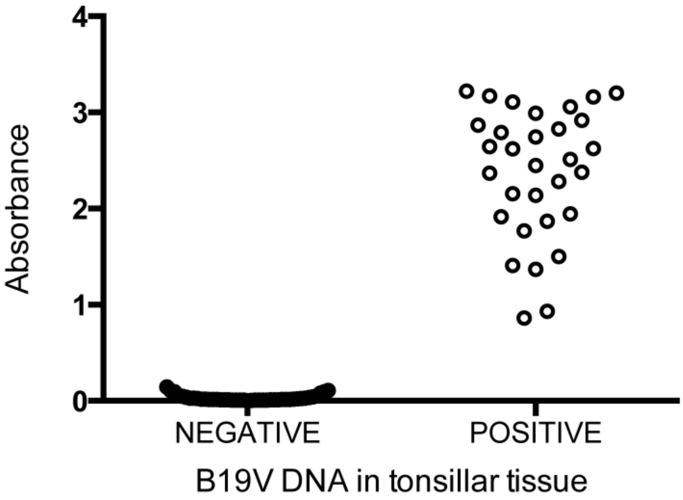

Supplementary Figure 3. B19V genoprevalence and seroprevalence. B19V genoprevalence (31 B19V-DNA positive vs 43 B19V-DNA negative individuals) in tonsillar tissues was strictly correlated to IgG serostatus in this study cohort.

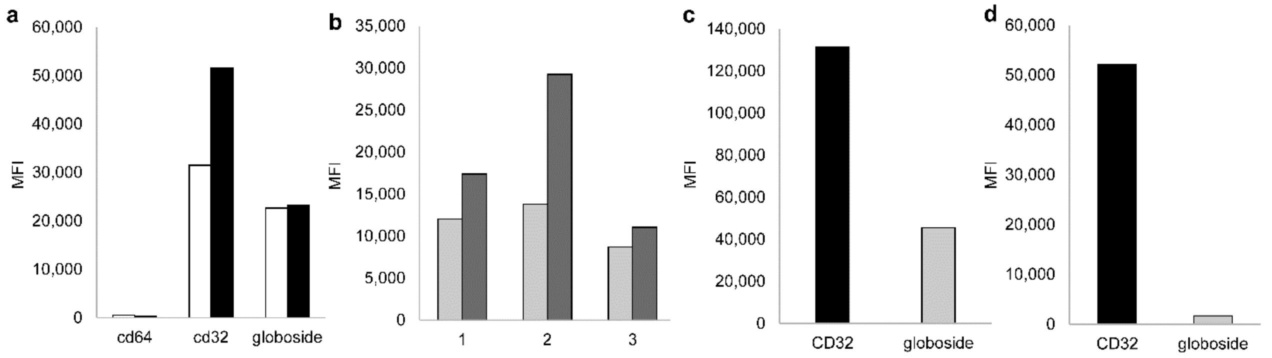

Supplementary Figure 4. Cell surface marker expression in cell lines and primary tonsillar B cells. **a**) Fc-receptors CD64 and CD32 and globoside expression levels in GM12878 (white bars) and Raji cells (black bars), as measured by flow cytometry; **b**) Globoside levels in total cell suspensions resulting from homogenization (light grey) or collagenase treated (dark grey) tissues from three seronegative individuals (*x* axis); **c**) CD32 (black) and globoside (light grey) expression in primary tonsillar B cells from one seronegative individual. **d**) CD32 (black) and globoside (light grey) expression in circulating B cells from venous blood of one seropositive staff member.

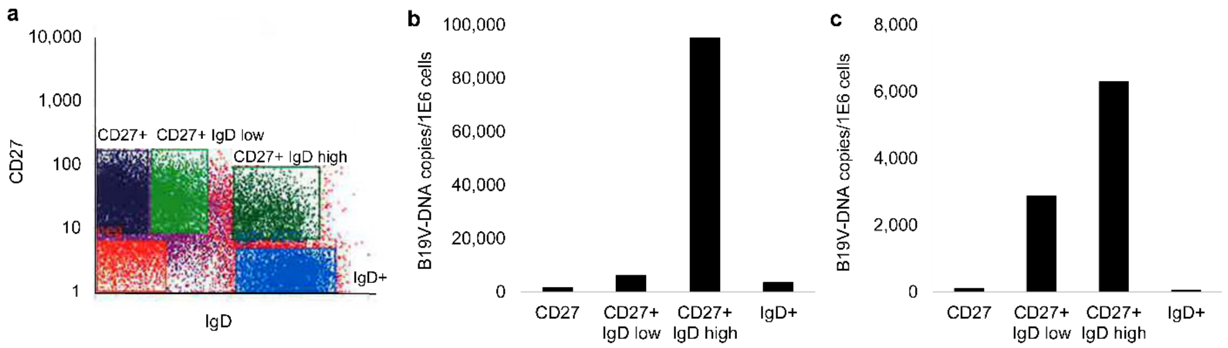

Supplementary Figure 5. Cell sorting of primary tonsillar B cells. a) Collagenase-treated tonsillar cell suspensions from 12 individuals were gated for CD19 expression and sorted according to their expression of CD27 (memory) or IgD (naive) cell surface markers. Represented are the four sub-populations collected from one of the individuals. **b**) and **c**) represent B19V-DNA copies in tonsillar B cell sub-fractions from two children of ages 8 and 6, respectively.

Supplementary Table 1. B19V genotype prevalence in the study cohort

Patient	Year of birth	Age[1]	B19V genotype	nt similarity to ref. seq.[2]
1	1946	69	**GT2**	100 %
2	1947	68	**GT2**	100 %
3	1962	52	**GT2**	100 %
4	1964	50	GT1	100 %
5	1968	46	**GT2**	100 %
6	1970	45	GT1	100 %
7	1974	40	GT1	100 %
8	1978	37	GT1	100 %
9	1978	37	GT1	100 %
10	1980	35	GT1	100 %
11	1980	34	GT1	100 %
12	1980	34	GT1	100 %
13	1980	34	GT1	100 %
14	1981	33	GT1	100 %
15	1981	33	GT1	98.1 %
16	1982	32	GT1	100 %
17	1984	31	GT1	99.4 %
18	1986	29	GT1	100 %
19	1986	29	GT1	100 %
20	1987	28	GT1	99.4 %
21	1987	27	GT1	100 %
22	1990	24	GT1	100 %
23	1992	22	GT1	100 %
24	1992	22	GT1	100 %
25	1994	20	GT1	99.4 %
26	1994	20	GT1	100 %
27	1995	20	GT1	100 %
28	1996	19	GT1	100 %
29	1998	17	GT1	100 %
30	1998	17	GT1	100 %
31	2006	8	GT1	100 %
32	2008	6	GT1	100 %
33	2009	6	GT1	100 %

[1] Age at the time of sampling
[2] Nucleotide similarity to the reference sequences FN598217.1 (genotype 1) and AY044266.2 (genotype 2).

Supplementary Table 2. EBV and B19V quantities in PBMCs and circulating B cells

Study subject	EBV DNA PBMC*	EBV DNA B cell*	B19V DNA PBMC*	B19V DNA B cell*	B19V IgG positive
1	1.15E+02	1.06E+03	0.00E+00	0.00E+00	Yes
2	0.00E+00	0.00E+00	0.00E+00	0.00E+00	Yes
3	0.00E+00	1.20E+02	0.00E+00	0.00E+00	Yes
4	3.26E+01	0.00E+00	0.00E+00	0.00E+00	Yes
5	0.00E+00	0.00E+00	0.00E+00	0.00E+00	Yes
6	7.99E+01	2.62E+02	0.00E+00	0.00E+00	Yes
7	0.00E+00	0.00E+00	0.00E+00	0.00E+00	Yes
8	0.00E+00	0.00E+00	0.00E+00	0.00E+00	No

* viral DNA copies / 1E6 cells

Supplementary Table 3. Sequences of primers and probes used in this study

PCR	Target	Oligo name	Sequence 5'-3'	References
**Pan-B19V qPCR**	Human parvovirus B19 *NS1*-gene	Fwd<br>Rev<br>Probe	CCACTATGAAAACTGGGCAATA<br>GCTGCTTTCACTGAGTTCTTCA<br>FAM-AATGCAGATGCCCTCCACCCAG-BHQ1	Toppinen *et al.* J Virol Methods 2015
**VP-qPCR**	Human parvovirus B19 *VP1*-gene	Fwd<br>Rev	CATGCCTTATCAYCCAGTARCAGT<br>AGGCCCAACATAGTTAGTACCG	Toppinen *et al.* Sci Rep 2015
**EBV-qPCR**	Epstein-Barr virus *BALF5*-gene	Fwd<br>Rev<br>Probe	CGGAAGCCCTCTGGACTTC<br>CCCTGTTTATCCGATGGAATG<br>FAM-TGTACACGCACGAGAAATGCGCC-BHQ1	Aalto *et al.* J Clin Virol 2003
**RNase P -qPCR**	Human *RNaseP* -gene	Fwd<br>Rev<br>Probe	GAGGGAAGCTCATCAGTGGGG<br>CCCTAGTCTCAGACCTTCCCAAG<br>FAM-AGTGCGTCCTGTCACTCCACTC-TAM	Toppinen *et al.* J Virol Methods 2015

BHQ=Black Hole Quencher
Y=C or T
R=A or G
